# COVID-19 Detection from Chest X-ray Images Using Feature Fusion and Deep Learning

**DOI:** 10.3390/s21041480

**Published:** 2021-02-20

**Authors:** Nur-A-Alam, Mominul Ahsan, Md. Abdul Based, Julfikar Haider, Marcin Kowalski

**Affiliations:** 1 Department of Computer Science and Engineering, Mawlana Bhashani Science and Technology University, Tangail 1902, Bangladesh; nuraalam.cse@diu-bd.net; 2 Department of Engineering, Manchester Metropolitan University, Chester St, Manchester M1 5GD, UK; j.haider@mmu.ac.uk; 3 Department of Electrical, Electronics and Telecommunication Engineering, Dhaka International University, Dhaka 1205, Bangladesh; based@kth.se; 4 Institute of Optoelectronics, Military University of Technology, Gen. S. Kaliskiego, 00-908 Warsaw, Poland; marcin.kowalski@wat.edu.pl

**Keywords:** COVID-19, X-ray image, deep learning, convolutional neural network (CNN), histogram-oriented gradient (HOG), watershed segmentation

## Abstract

Currently, COVID-19 is considered to be the most dangerous and deadly disease for the human body caused by the novel coronavirus. In December 2019, the coronavirus spread rapidly around the world, thought to be originated from Wuhan in China and is responsible for a large number of deaths. Earlier detection of the COVID-19 through accurate diagnosis, particularly for the cases with no obvious symptoms, may decrease the patient’s death rate. Chest X-ray images are primarily used for the diagnosis of this disease. This research has proposed a machine vision approach to detect COVID-19 from the chest X-ray images. The features extracted by the histogram-oriented gradient (HOG) and convolutional neural network (CNN) from X-ray images were fused to develop the classification model through training by CNN (VGGNet). Modified anisotropic diffusion filtering (MADF) technique was employed for better edge preservation and reduced noise from the images. A watershed segmentation algorithm was used in order to mark the significant fracture region in the input X-ray images. The testing stage considered generalized data for performance evaluation of the model. Cross-validation analysis revealed that a 5-fold strategy could successfully impair the overfitting problem. This proposed feature fusion using the deep learning technique assured a satisfactory performance in terms of identifying COVID-19 compared to the immediate, relevant works with a testing accuracy of 99.49%, specificity of 95.7% and sensitivity of 93.65%. When compared to other classification techniques, such as ANN, KNN, and SVM, the CNN technique used in this study showed better classification performance. K-fold cross-validation demonstrated that the proposed feature fusion technique (98.36%) provided higher accuracy than the individual feature extraction methods, such as HOG (87.34%) or CNN (93.64%).

## 1. Introduction

The COVID-19 is a deadly disease caused by the newly recognized coronavirus. In December 2019, coronavirus (SARS-COV-2) infected the human body for the first time, and it can spread principally among humans through the droplets formed by the infected persons when they speak, cough or sneeze [1,2,3,4,5,6]. As the droplets are too heavy to travel far, they cannot spread person-to-person without coming in close contact [7]. Although the exact time is not yet known, a new study has estimated that the COVID-19 can be viable in the air for up to 3 hours, on copper for 4 hours and up to 72 hours on plastic and stainless steel. However, the exact answers to these questions are still not agreed upon by the general health research community and currently under investigation. COVID-19 attacks the lung and damages the tissues of an infected person. At the early-stage, some people may not find any symptoms where most of the people had fever and cough as the core symptoms. Other secondary symptoms could be body aches, sore throat, and a headache could be all possible.

At present, COVID-19 disease is increasing daily due to the lack of quick detection methods. All over the world, a huge number of people died of this disease in 2020. The respiratory tract and lungs are the media where the virus can spread easily. As a result, inflammation occurs, and air sacs can be filled with fluid and discharge. The process is responsible for creating an obstacle in oxygen intake. Quick and accurate detection of the virus is a major challenge for doctors and health professionals around the world in order to reduce the death rate caused by this virus.

**Figure 1:**
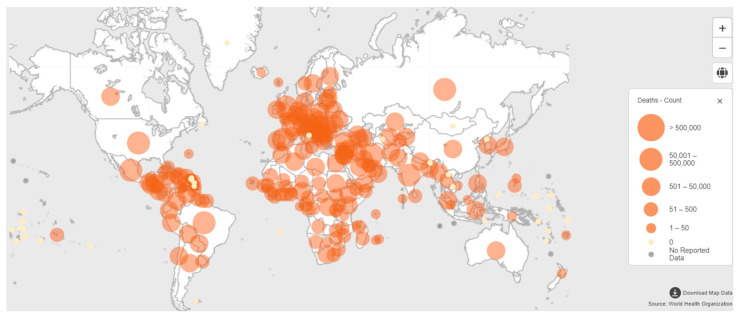
Map for coronavirus-related deaths across the globe reported to WHO on 7 January 2021 (source: World Health Organization, WHO).

Due to the global climate changes, people have already been suffering from many other diseases, and the impact created by the COVID-19 is immeasurable. Currently, the virus has spread to almost every country in the world [8]. Recently, all over the world, America, South-East Asia, and Europe have the uppermost number of confirmed COVID-19 cases (Figure 1). On 7 January 2021, more than 85,929,428 confirmed cases of the virus and 1,876,100 deaths were reported by World Health Organization (WHO) due to the disease [8]. At present, further research on an effective screening process is required for diagnosing the virus cases and segregating the affected people. Health professionals and scientists of many countries in the world are attempting to improve their treatment plan and capacity of test through implementing multifunctional testing to stop spreading the virus and for protecting themselves from the deadly virus.

Currently, a number of countries have developed vaccines for the COVID-19. Among them, the vaccines developed by Pfizer (USA), AstraZeneca (UK) and Moderna (USA) have been accepted and used in the USA, UK, and many countries in Europe. Based on the clinical-trial data, it has been claimed that the three popular vaccines have achieved the target of 50% efficacy and safe to use without any serious side effects [9,10]. The Pfizer vaccine is required to store at -70 °C temperature. This low-temperature storage makes it challenging to transport and store all over the world, particularly in underdeveloped countries. However, the AstraZeneca vaccine requires regular fridge temperature, which will be easier for both carrying and storing worldwide. More recently, the vaccine developed by Sinovac Life Sciences in China has been approved in many countries globally, including Brazil, Indonesia, Colombia, Turkey, Chile, Uruguay and Laos [11]. Furthermore, Sputnik V was developed by Gamaleya Institute, Russia and is currently being used in Belarus, United Arab Emirates, Saudi Arabia, India and Iran [12]. Furthermore, mass vaccination worldwide still remains a huge logistical challenge [13]. Still, large-scale manufacturing is required to produce the vaccine for covering people all over the world. Further research is required on how long the protection lasts and to find out the effectiveness of the vaccines, particularly against new variants of viruses, which are currently detected in the UK, South Africa, Brazil, and Portugal.

Deep learning with CNN also has been employed in disease diagnosis, such as cancer, via image classification. For example, Li and Shen [14] have proposed two fully convolutional residual networks to produce segmentation, feature extraction and classification result from skin lesion images. A lesion index calculation unit was used to refine the classification results. The results achieved from the deep learning frameworks showed good accuracies (0.912) in cancer diagnosis. Liao et al. [15] proposed a multitask deep learning (MTDL) method to improve diagnosis performance for twelve types of cancer, while their expression data were not adequate. Their experiments showed that the proposed method learned from the aggregation of the expression data for the twelve types of cancer to diagnose cancer accurately. However, the authors did not compare the performance with existing similar works. Yoo et al. [16] have developed an automated CNN-based method for detecting prostate cancer using diffusion-weighted magnetic resonance imaging (DWI) collected from 427 patients. First-order statistical features were extracted from slice-level probabilities, which were used to compile slice-level classification results into the patient level. The proposed method was tested on 108 patients and found good results for both slice level and patient level. However, their system could use 3D CNNs and other deep learning methods to obtain better cancer diagnosis. Esteva et al. [17] have demonstrated skin cancer classification by pre-trained Inception V3 CNN model on 129,450 clinical skin cancer images and 3374 dermatoscopic images. The CNN was trained end-to-end from the images using pixels and disease labels as inputs. The overall CNN accuracy was found as 72.1 ± 0.9% (mean ± s.d.), whereas accuracies of 65.56% and 66.0% were achieved by two dermatologists on a subset of the validation set.

Recently, the reverse transcriptase-polymerase chain reaction (RT–PCR) diagnostic method is found to be effective in detecting the virus. However, the method has some drawbacks, including longer detection time and lower detection rate of the virus. Strict requirements in the laboratory and diverse characteristics of the testing could be attributed to the drawbacks [18,19]. Researchers are working on overcoming the limitations of RT–PCR testing to enhance diagnosing and detection of the COVID-19. According to the recommendations by WHO provided in October 2020, chest imaging examination is an effective method for the detection of clinical symptoms of people who have been affected and recovered from the virus [20]. Furthermore, other diagnostics tests are also suggested, including ultrasound, X-rays and MRI of the chest and computed tomography (CT) and needle biopsy of the lung. At present, chest X-ray is extensively used for the detection of the COVID-19 cases compared to the CT image as it takes longer for imaging, and CT scanners are not available in many underdeveloped countries. In addition, CT imaging is highly costly, and pregnant women and children may face health risks due to its high radiation [21]. On the contrary, X-ray imaging has played a great role in many medical and epidemiological cases due to its wider availability [22,23]. Chest X-ray is promising for emergency cases and treatment due to its operational speed, cost and simplicity for the radiologists. However, in prior research, some inconsistencies were observed for the chest X-ray images taken from people affected by the COVID-19 [24].

In the past, artificial intelligence (AI) techniques were employed to successfully diagnose Pneumonia either from chest X-ray images or CT [25,26,27]. The classification methods employed vary from Bayesian function to convolutional neural network (CNN). More recently, CNN has been found to be useful and effective in identifying COVID-19 via image classification. CNN consists of multilayer neural networks, which are highly capable of recognizing the image patterns without conducting diverse preprocessing of the images. Although several CNN models, including AlexNet, Resnet50, VGG16, VGG19, are available, VGG19 demonstrates better performance for the COVID-19 classification [18,26,27,28].

The proposed work here provides an intelligent machine learning architecture in order to detect COVID-19 disease using chest X-ray images. The method proposes a novel fusion of features extracted by histogram-oriented gradient (HOG) and CNN and classification by CNN. Furthermore, a modified anisotropic diffusion filtering (MADF) technique was applied to eliminate multiplicative speckle noise from the test images. The watershed segmentation technique was used to identify the fractured lung regions, which could further provide evidence for the COVID-19 attacked lungs.

The remaining sections of this research work are formulated as follows: A comprehensive study on the classification of chest X-ray images is presented in Section 2. Section 3 describes the system architecture used to identify COVID-19 from X-ray image datasets by classifying it into the COVID-19 and other classes. Section 4 presents the results with analysis achieved from this work. Then, Section 5 discusses validation, comparative performances, and limitations of the proposed system in detecting COVID-19. Finally, conclusions drawn from this research are presented in Section 6.

## 2. Related Literature

In recent months, researchers have investigated and analyzed chest X-ray images using deep learning algorithms to detect COVID-19. First, the images are preprocessed using the CNN technique for extracting better features, which are fed in deep learning algorithms for image classification. Ahammed et al. [29] proposed a deep neural network-based system where CNN provided high accuracy (94.03%). The authors trained the system with normal, pneumonia and COVID-19 patient’s chest X-ray images. The limitation of the work was that a dataset with only 285 images was used for developing the system, and this small number of data was not perfect for training a deep learning-based system for the COVID-19 prediction.

Chowdhury et al. [30] worked with chest X-ray images to develop a novel framework named PDCOVIDNet based on parallel-dilated CNN. In the proposed method, the authors used a dilated convolution in the parallel stack that could capture and stretch necessary features for obtaining a detection accuracy of 96.58%.

Abbas et al. [31] proposed and validated a deep convolutional neural network called decompose, transfer, and compose (DeTraC) to detect COVID-19 patients from their chest X-ray images. They proposed a decomposition mechanism to check irregularities from the dataset by investigating class boundaries for obtaining a high accuracy (93.1%) and sensitivity (100%).

Azemin et al. [32] used a deep learning method based on the ResNet-101 CNN model. In their proposed method, thousands of images were used in the pre-trained phase to recognize meaningful objects and retrained to detect abnormality in the chest X-ray images. The accuracy of this method was only 71.9%.

El-Rashidy et al. [33] introduced a framework consisted of three layers: patient layer, cloud layer and hospital layer. A set of data was collected from the patient layer using some wearable sensors and a mobile app. A neural network-based deep learning model was used to detect COVID-19 using the patient X-ray images. The proposed model achieved 97.9% accuracy and 98.85% specificity.

Khan et al. [34] developed a new architecture for the diagnosis of X-ray images as the COVID-19 or normal using pre-trained deep learning models like ResNet50, VGG16, VGG19 and DensNet121, where VGG16 and VGG19 showed the best accuracies. The proposed model consisted of two phases like preprocessing and data augmentation, and transfer learning, and finally showed 99.3% accuracy.

In the proposed model by Loey et al. [35], three deep transfer models like AlexNet, GoogleNet and ResNet18 were employed on a dataset of 307 images with four different types of classes: COVID-19, normal, pneumonia bacterial and pneumonia virus. The research work was distributed into three scenarios to reduce memory consumption and execution time. At the last deep transfer model, GoogleNet achieved 100% testing accuracy and 99.9% validation accuracy.

Minaee et al. [36] reported a deep learning-based framework to detect COVID-19 from chest X-ray images using four tuning models like ResNet18, ResNet50, SqueezeNet and DensNet-121. The proposed method took advantage of data augmentation to create a transformed version of the COVID-19 images, which increased the number of samples and finally achieved 98% sensitivity and 90% specificity.

Sekeroglu et al. [37] developed a model using deep learning and machine learning classifiers where a total of 38 experiments was conducted by CNN for the detection of the COVID-19 using the chest X-ray images with high accuracy. Among them, 10 experiments were performed using 5 different machine-learning algorithms, and 14 experiments were carried out by the state-of-the-art pre-trained network for transfer learning. The system demonstrated 98.50% accuracy, 99.18% specificity and 93.84% sensitivity. They concluded that the system developed by CNN was capable of achieving COVID-19 detection from a limited number of images without any preprocessing and with minimized layers.

Wang et al. [38] developed a model using ResNet-101 and ResNet-151 with fusion effects to enhance their weight ratio dynamically. Classification of the chest X-ray images was carried out based on three classes, such as normal, COVID-19 and viral pneumonia. Performance accuracy of 96.1% was achieved during the testing phase.

Yoo et al. [39] applied chest X-ray radiography (CXR) images to classify using a deep learning-based decision-tree classifier for detecting COVID-19. This classifier compared three binary decision trees based on the PyTorch frame. The decision tree classified CXR images as normal or abnormal, where the third decision tree achieved an average accuracy of 95%.

Khalifa et al. [40] developed a classification approach for the treatment purposes of coronavirus on a single human cell-based on treatment type and treatment concentration level using deep learning and machine learning (ML) methods. Numerical features of the data sets were converted to images for building the DCNN model. The testing accuracy of treatment classification obtained by the model was as high as 98.05% compared to the other traditional ML methods, including support vector machine (SVM) and decision tree (DT). However, the proposed DCNN model showed less testing accuracy (98.2%) compared to the DT (98.5%) for the prediction of treatment concentration level. Deep transfer models (i.e., Alexnet) have not been employed in their study.

Wang et al. [41] have developed a transfer learning method (Xception model) using deep learning models for diagnosing COVID-19. The proposed method showed 96.75% diagnostics accuracy. Furthermore, Deep features and machine learning classification (Xception + SVM) were also employed to develop an efficient diagnostic method for improving the accuracy of the Xception model by 2.58%. From the result, the authors claimed that their proposed method attained higher classification accuracy and efficient diagnostic performance of the COVID-19. However, the authors have not compared their results with the existing similar works.

Sahlol et al. [42] proposed an improved hybrid classification approach using CNNs and marine predators algorithm for classifying COVID-19 images, which were obtained from international cardiothoracic radiologists. Inception architecture of CNNs was employed to extract features, and a swarm-based marine predators algorithm was used to select the most relevant features from the images. However, the research work did not consider any fusion approach to improve the classification and feature extraction of the COVID-19 images.

Most of the reported work in the literature has used chest X-ray images to diagnose COVID-19, and this highlights the importance of chest X-ray image analysis as an indisputable tool for doctors and radiographers. However, imbalance in data manipulation and lack of necessary extracted features from the images sometimes cannot provide expected accuracy in the classification result. To overcome these limitations, this work proposed fusion of features extracted by HOG and CNN and classify using CNN for improving the detection accuracy of the COVID-19.

## 3. Proposed Methodology

### 3.1. System Architecture

The proposed system considered input of the X-ray images to identify COVID-19. First of all, this system converted images from RGB to grayscale and identified the region of interest (ROI) by removing the unwanted regions. Furthermore, the system considered two feature extractors: histogram-oriented gradient (HOG) and CNN. First, the HOG technique was used to extract a feature vector from the X-ray COVID-19 dataset. Then the CNN method was used to extract another feature vector from the same images. These two features were fused and used as the input to train the classification model. The number of features extracted by one technique was not large enough to accurately identify COVID-19. However, the fusion approach of extracting features by two different techniques could provide a large number of features for accurate identification. Fusion was considered as a concatenation between the two individual vectors in this context.

Speckle-affected and low-quality X-ray images along with good quality images were used in our experiment for conducting tests. If training and testing are performed with only selected good quality X-ray images in an ideal situation, the output accuracy may be found higher. However, this does not represent a real-life scenario, where the image database would be a mix of both good- and poor-quality images. Therefore, this approach of using different quality images would test how well the system can react to such real-life situations.

**Figure 2:**
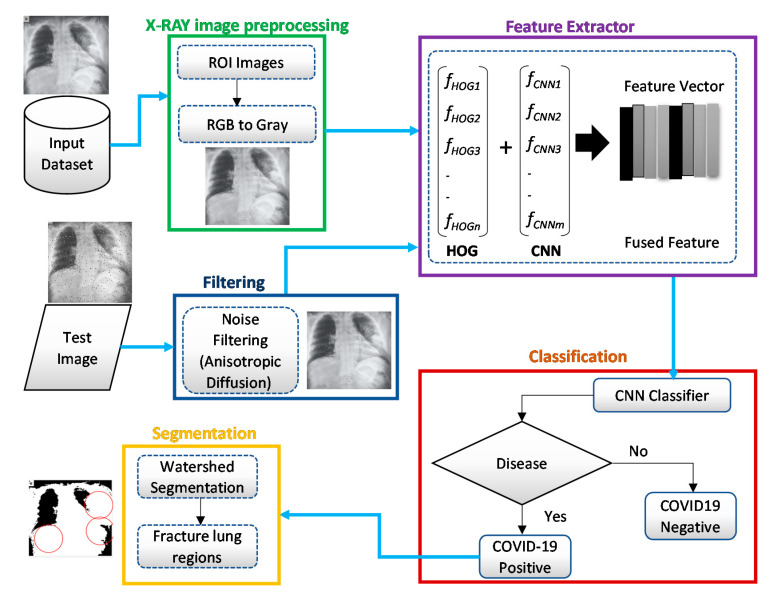
Overview of the proposed intelligent system architecture for identifying COVID-19 from chest X-ray images.

A modified anisotropic diffusion filtering technique was employed to remove multiplicative speckle noise from the test images. The application of these techniques could effectively overcome the limitations in input image quality. Next, the feature extraction was carried out on the test images. Finally, the CNN classifier performed a classification of X-ray images to identify whether it was COVID-19 or not. Figure 2 shows the basic steps of the proposed system architecture, which is also represented by Algorithm 1.

### 3.2. Dataset Used

The chest X-ray images of the patients were acquired and stored in a commonplace. The images were categorized as either COVID-19-positive or negative as a reference to evaluate the performance of the intelligent system. In this work, three standard datasets were employed to validate the system’s performance.

(1) The benchmark data set [43] used in our experimental evaluation consisted of two main categories with 819 COVID-19-positive and 1341 normal chest X-ray images;

(2) Cohen’s data set [44] contained a total of 660 images with 390 positive COVID-19 X-ray images;

(3) Another publicly available [45] dataset was used with 770 images of the COVID-19 and 1500 normal images.

The databases contained various sizes of images ranging from 512 × 512 pixels to 657 × 657 pixels. The acquired images were in both grayscale and RGB formats, and the RGB images were converted to grayscale images. Any feature extraction method can easily detect features from grayscale images compared to the images in other formats. To convert RGB to grayscale image, Equation (1) is used for calculating the grayscale value (I) by forming a weighted sum of the monochrome colors, red (R), green (G), and blue (B).

I = (Wr. R) + (Wg. G) + (Wb. B)(1)

Wr, Wg, and Wb are the weights of red, green, and blue colors, respectively, with a value of 0.30, 0.59, and 0.11, summing to a total equal to 1.

Furthermore, the data formats of the images included png and jpeg with bit depths of 8-bit (grayscale) and 24-bit (RGB). As the image size, format and bit depth were different in the databases, they were converted to a size of 224 × 224 pixels with 8-bit grayscale images and saved in png format.
**Algorithm 1** Proposed Algorithm for COVID-19 Detection
Input: COVID-19 Chest X-ray image dataset (D) with resize image (M) Extraction: Extract Feature Matrix (f). CNN Feature Vector (Fc). Step 1: Initialize $F_{c}$ ≥ $M_{i}.i=1$ Step 2: Extract each image feature $D\left(i,1,570\right).$ Step 3: $F_{c}$
$\left(i,1\right)$ = $M\left(x,1\right)$ + $F_{c}\;\left(i,1\right).$ Step 4: $F_{c}$ = overall CNN features. Histogram Oriented Gradient (HOG). Step 1: Initialize. $H_{0}=Low\;pass\;output$,$H_{1}=Band\;pass\;output$. Step 2: HOG $\left(i,1\right)$ = $H_{0}$
$\left(i,1\right)$ + $H_{1}$
$\left(i,1\right)$. Step 3: HOG = overall Histogram Oriented Gradient Fusion of features in Vector (V). $Training\;feature\;\left(V\right)=[F_{c}\;,\;HOG]$**.** $test\_image=imread\left(img\right)$. Extract test feature (T) = repeat step 1, 2 from test_image. $result\;\left(i\right)=classify\;\left(training\;feature,\;T\right)$. Output: $result\;\left(i\right)=\;COVID19\;Positive\;or\;Normal.\;$


**Figure 3:**
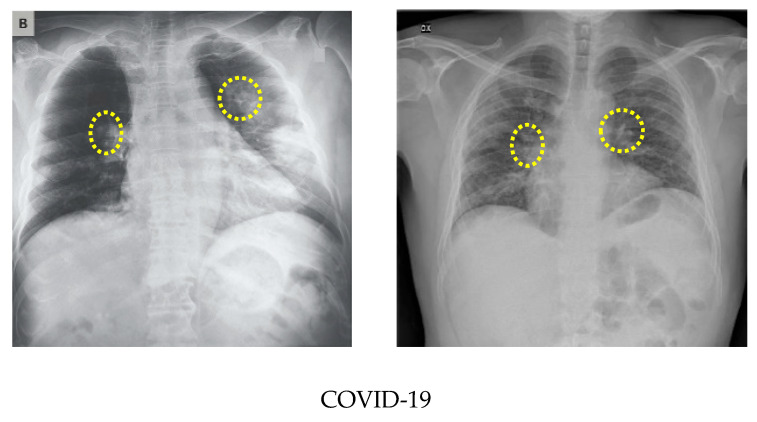

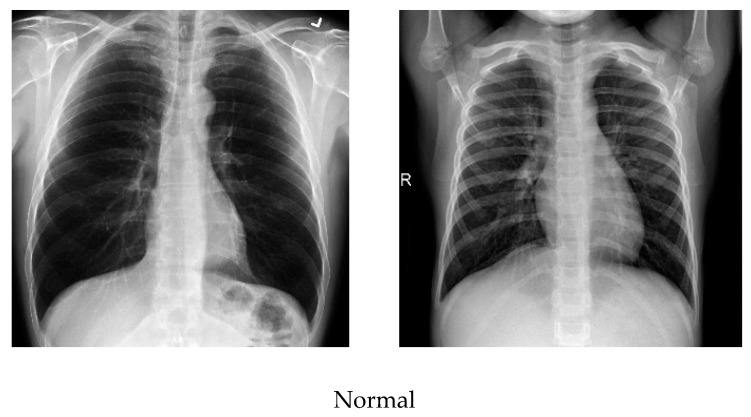
Comparison of the COVID-19 and normal X-ray images.

As the number of images available in the open repository was limited, the images from all three databases were combined to create a database for this work. The training and testing stages used both classes: COVID-19-positive and COVID-19-negative. The COVID-19-positive class contained 1979 images, and the COVID-19-negative class contained 3111 images. All data sets were divided into 0.8 portions and 0.2 portions for the training and testing, respectively. A computer having 64-bit windows, 8 GB RAM, Intel Core i5 CPU with a processing speed of 2.60 GHz was used for performing all training and testing. MATLAB 2019b was used to execute all necessary experiments. The experimentation times are computed with a GPU hardware configuration of GEFORCE RTX 2070 super. Figure 3 illustrates a comparison of the COVID-19 and normal chest X-ray images. In general, similar to pneumonia, the density of the lungs is increased in the case of the COVID-19, which causes whiteness in the lungs on radiography. An experienced radiologist can confirm the disease by the appearance of a ground-glass pattern (ground-glass opacity) due to the increased whiteness [46].

### 3.3. Data Preprocessing

Image processing is an important step to achieve meaningful information and accurate classification by removing noisy or deformed pixels from each image. First, the images were converted from RGB to grayscale using the MATLAB tool and resized to 224 × 224 pixels to be made ready as input to the system.

**Figure 4:**
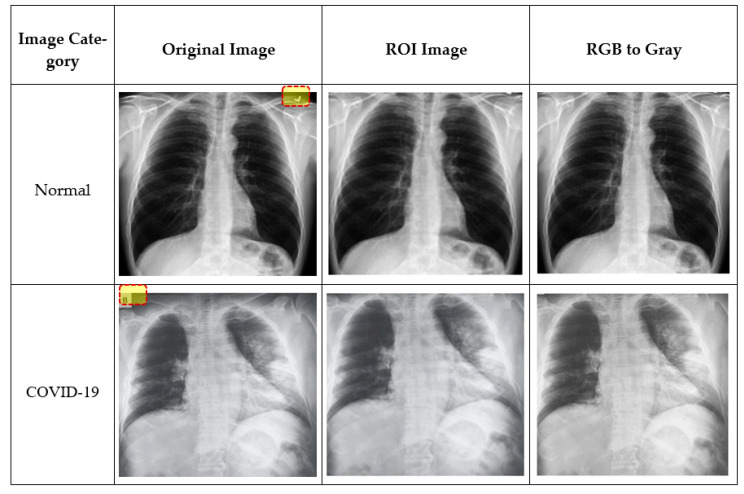
Proposed preprocessing stages: original image, the region of interest (ROI) image and 24-bit (RGB) to gray.

To eliminate superfluous text and machine annotations around images, the region of interest (ROI) was extracted for training and testing. In order to obtain meaningful information, the ROI on the chest X-ray images was defined by an area covering mainly the lung region. First, an ROI is defined by a rectangle, and a mask is created from the rectangle. Using logical indexing, the area outside the ROI was set to zero, and the extracted portion is displayed. Figure 4 illustrates example images at different preprocessing stages. For example, unnecessary symbols (tick mark in normal image) or text (B in the COVID-19 image) in the original images were removed at the ROI stage. As the images used in this study were collected from three different sources, they might differ in quality, size, or inherent noise. Therefore, the preprocessing approaches employed would normalize all the images such that they were independent of their origin, and the image size effect on the system’s performance could be avoided [39].

### 3.4. Modified Anisotropic Diffusion Filtering (MADF)

Filtering techniques preserve useful information while filtering any noise within an image. To extract the meaningful features of any noisy image, the information-preserving filtering techniques are most applicable [21]. During the testing phase, speckle-affected test images were used to assess filtering performance. Anisotropic diffusion filtering can preserve and enhance edge information while subduing any noise [24,47]. The gradient operator detects the edge information along with noise [48]. This technique finds the gradient changes in noise for strong speckle and low contrast images, which may go beyond the gradient of edge. These changes destruct the edge information than the noise, which provides less accuracy in filtering results. Similarly, speckle reducing anisotropic diffusion (SRAD) cannot fully preserve the edge information due to image over-smoothing [49]. Oriented-based non-local means (OBNLM) fails to hold detailed information and is affected by moving noise [27]. Anisotropic diffusion with memory-based speckle statistic (ADMSS) is sharper in white pixels [50].

Modified anisotropic diffusion filtering (MADF) was proposed for this work in order to preserve detailed information while reducing noise and distortion from the images. This filtering technique performs better than the other filtering methods due to its capability in eliminating multiplicative speckle noise in plane regions. The proposed method uses correlation and kurtosis values of noise to hold the useful edge information. In Equation (2), I_o_ is a noisy image comprised of speckle-noise n and the original image I [27,49]. The noise part is denoted by Equation (3), where G is noise intensity and calculated from image properties in MATLAB. The mean of noise intensity is µ, which is calculated by Equation (4). Kurtosis k is calculated using Equation (5). The correlation between the image class and noise class should be minimum, which is the iteration stopping condition. This speckle suppression process continues until the noise part of the image is close to the Gaussian value. In this situation, the kurtosis value should be zero. The iteration cutoff is defined when the kurtosis value falls below 0.001 (Equation (6)), indicating a low speckle with better edge preservation. As soon as the correlation between image class and noise class is the least, the iteration will be stopped. Equation (7) calculates the correlation of image intensities ($\rho_{I\;}$) and Equation (8) calculates the correlation of noise intensities ($\rho_{G\;}$). The proposed filtering will get the optimal result when $\rho_{I\;}$ and $\rho_{G\;}$ show minimum deviance.

(2) $$I_{0}\;=\;I.n$$

(3) $$n\;=\frac{I-G}{\surd G}\;$$

(4) $$µ\;=\;\frac{\sum_{i=1}^{N}G_{i}}{N}$$

(5) $$k=\;\frac{\frac{1}{N}\;\sum_{i\;=\;0}^{N}{\left(G-µ\right)}^{4}}{{\left[\frac{1}{N}\;\sum_{i\;=\;0}^{N}{\left(G-µ\right)}^{2}\right]}^{2}}\;-\;3$$

(6) $$abs\left(n-k\right)\leq 0.001$$

(7) $$\rho_{I}\;=\;\frac{\sum_{i\;=\;0}^{M-1}\sum_{j\;=\;0}^{N-1}i.j.p_{I}\left(i,j\right)-µI_{x}µI_{y}}{\frac{\sum_{i\;=\;1}^{N}\left(I_{ix}-\mu_{Ix}\right)(I_{iy}-\mu_{Iy})}{N}}$$

(8) $$\rho_{G\;}=\;\frac{\sum_{i\;=\;0}^{M-1}\sum_{j\;=\;0}^{N-1}i.j.p_{G\;}\left(i,j\right)-µG_{x}µG_{y}}{\frac{\sum_{i\;=\;1}^{N}\left(G_{ix}-\mu_{Ix}\right)(G_{iy}-\mu_{Iy})}{N}}$$

**Figure 5:**
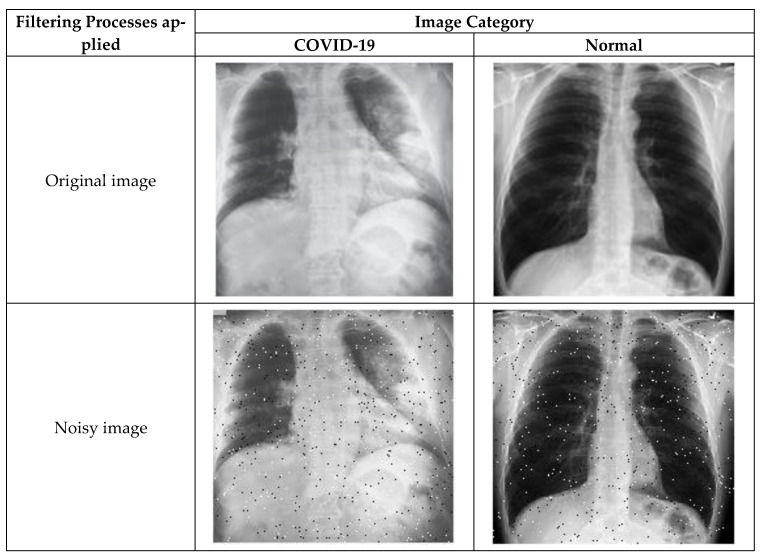

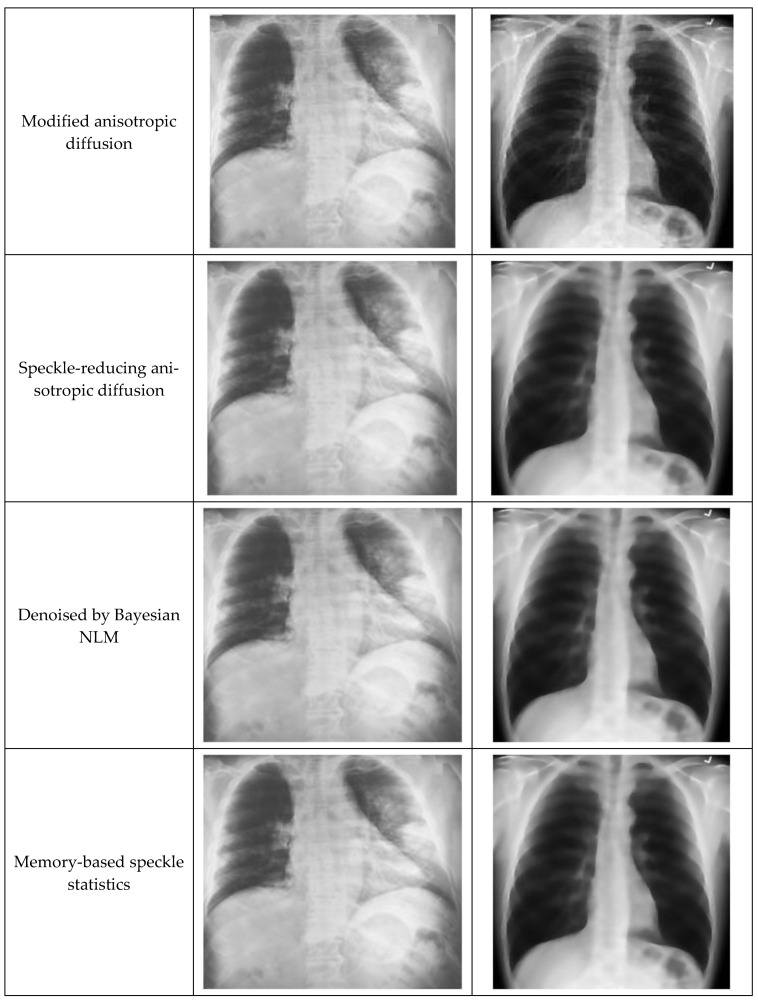
Illustration of images after applying different anisotropic diffusion techniques.

Figure 5 shows an example of original images and a comparison of different Anisotropic Diffusion techniques. It was very clear that the edge preservation capability of the proposed MADF technique was much better than the other techniques.

Upon applying MADF, the images are divided into multiple small parts of images, which is called a gradient. Then these parts are filtered one-by-one. After filtering all parts, they are merged together. In general, the anisotropic diffusion filtering method removes all noises and edge information from the images. If this method is applied to the image with many iterations, the edge information in the images is removed. If the k value is 0, then all image features or in-formation is removed. Therefore, filtering continues up to a standard k value of 0.001 in order to obtain an appropriate filtered image. In general, it is quite difficult to ex-tract appropriate features from the blur images, whereas the MADF method solves this issue.

### 3.5. Feature Extractor

#### 3.5.1. Histogram-Oriented Gradient (HOG) Feature Extractor

Histogram-oriented gradient (HOG) system extracts features by using a selective number of histogram bins [51]. For extracting HOG features, the proposed system used a higher number of histogram bins on different regions of the images. First, the input image was scaled to 64 × 128 pixels and converted into a grayscale image. The gradient for every pixel in the image was calculated using Equations (9) and (10).

(9) $$d_{x}=I\left(x+1,y\right)-I\left(x,y\right)$$

(10) $$d_{y}=I\left(x,y+1\right)-I\left(x,y\right)$$

where d*_x_* and d*_y_* are the horizontal and vertical gradient, respectively, and *I (x, y)* is the pixel value at *(x, y)* position. The gradient orientation, θ, is then calculated using Equation (11):

(11) $$\theta \left(x,y\right)=\;\tan^{-1}\frac{d_{y}}{d_{x}}$$

**Figure 6:**
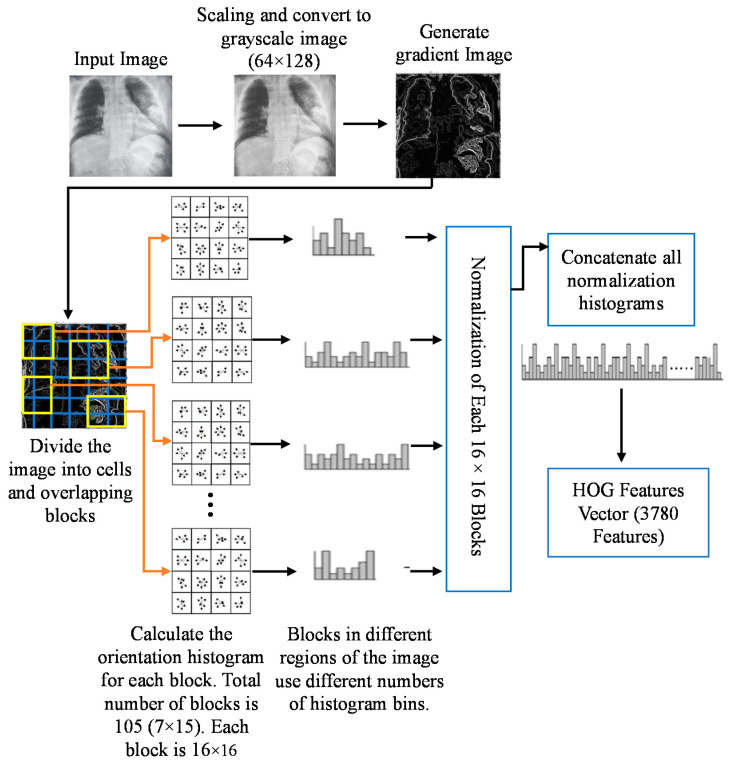
Basic flow of histogram-oriented gradient (HOG) feature extraction algorithm.

The basic flow of the HOG feature extraction algorithm is shown in Figure 6.

The gradient image was divided into cells size of 8 × 8 pixels to generate the histogram. As a result, a 9 × 1 matrix for each cell was obtained. Then the gradients were normalized by taking 16 × 16 blocks. Four 8 × 8 cells were combined to create a 16 × 16 block. Therefore, four 9 × 1 matrices or a single 36 × 1 matrix could be obtained. Mathematically, for a given vector V (Equation (12)), the root of the sum of squares (Equation (13)) can be calculated.

V = [a1, a2, a3, ….a36](12)

(13) $$k=\sqrt{{\left(a1\right)}^{2}+\;{\left(a2\right)}^{2}+\;{\left(a3\right)}^{2}+\;\ldots .\;{\left(a36\right)}^{2}}$$

After dividing all the values in vector V with the k value (Euclidian norm or the length of the vector), the resultant would be a normalized vector of size 36 × 1 as defined by Equation (14).

(14) $$Normalised\;Vector=\left(\frac{a_{1}}{K},\frac{a_{2}}{K},\frac{a_{3}}{K},\frac{a_{4}}{K}\ldots \ldots \ldots \frac{a_{36}}{K}\right)$$

In this case, a total of 105 (7 × 15) blocks of 16 × 16 can be obtained. Each of these 105 blocks will have a matrix of 36 × 1 as features. Thus, the total number of features for the image would be 105 × 36 × 1 = 3780.

#### 3.5.2. CNN Based Feature Extractor and Classification

Image processing, particularly features extraction by employing CNN, is an important research topic in computer science [52]. An experiment was conducted using scratch and pre-trained CNN models in this proposed work. The results achieved by the scratch model were not satisfactory; however, the pre-trained model showed good performance.

**Figure 7:**
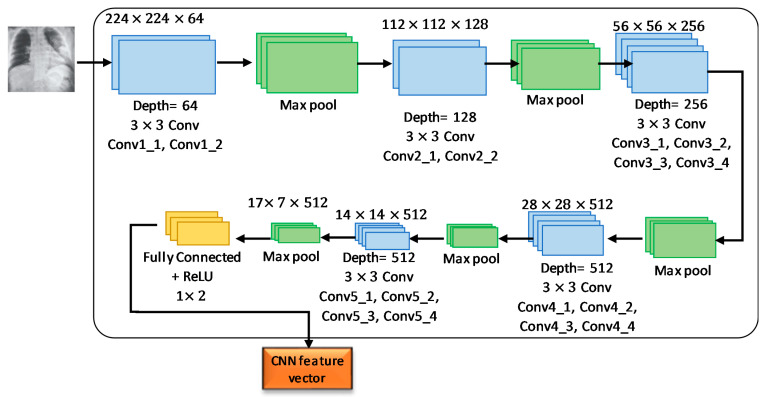
Illustration of image feature extraction by VGG19 pre-trained convolutional neural network (CNN) model.

VGG19 model (pre-trained) was fine-tuned to suit as a feature extractor for the experimental dataset used in this study. A VGGNet consisting of 19-layer was used to develop this network model. Experimental trial asserted that VGG19 showed better performance compared to VGG16, scratch model and other deep learning models, including ResNet50 and AlexNet. The VGG19 model was developed using sixteen convolution layers with three fully connected layers (Figure 7). A nonlinear ReLU was used in the activation function for getting the output of convolution layers, whereas the convolution part was split by five consecutive max-pooling layers. Two convolution layers were used to develop the first and second subregions, where the depth of the layers was 64 and 128. Furthermore, four consecutive convolution layers were used to build the remaining three subregions where the depth of the layers were 256, 512, and 512, respectively. Afterward, Pooling layers were employed to decrease the learnable parameter. The last layer of the proposed VGG19 model helped in obtaining the feature vector, whereas 1024 and 512 neurons existed in the two hidden layers placed before the feature collection layer. For reducing the overfitting during the implementation of the fine-tuned model, L2 regularization was employed after each fully connected layer. The CNN-based VGG19 models provide 4096 appropriate features.

### 3.6. Feature Fusion and Classification

Data fusion was applied in several machine learning and computer vision applications [53]. Particularly, feature fusion can combine more than one feature vector. Two feature extractors provide a feature vector of 1 × 4096 and 1 × 3780. The feature selection process was mathematically explained by Equations (15)–(17) [54]. Equation (15) and Equation (16) represent features extracted by HOG and CNN, respectively. The extracted feature vectors are combined by concatenation and represented by Equation (17).

(15) $$f_{HOG\;1\times n}=\left\{\;HOG_{1\times 1},\;HOG_{1\times 2},\;HOG_{1\times 3}--------HOG_{1\times n}\right\}$$

(16) $$f_{VGG19\;1\times m}=\left\{\;VGG19_{1\times 1},\;VGG19_{1\times 2},\;VGG19_{1\times 3}-----VGG19_{1\times m}\right\}$$

(17) $$Fused\;{\left(features\;vector\right)}_{1\times q}^{cat}\;=\;\left\{f_{HOG\;1\times n},\;f_{VGG19\;1\times m}\right\}$$

Then the features extracted by HOG and CNN are fused with 7876 features. 1186 score-based features were selected out of 7876 features based on maximum entropy. When the value of i = 1, it recalls HOG features and when i = 2, it recalls VGG19 features and finally adds them together. For the purpose of selecting optimal features, entropy was employed considering score values. The probability of features and entropy is defined by Equations (18) and (19). The final selected features were fed to the classifiers in order to identify COVID-19 images.

(18) $$B_{He}=\;-NHe_{b}\sum_{i\;=\;1}^{n}p\left(f_{i}\right)$$

(19) $$F_{select}\;=\;B_{He}\left(\max\left(f_{i\;},1186\right)\right)$$

**Figure 8:**
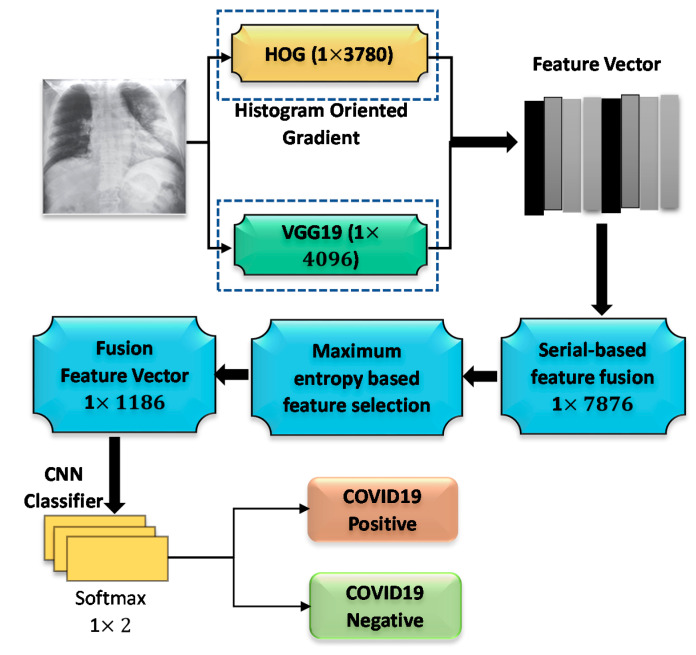
The proposed fusion steps for the features extracted by HOG and CNN.

Where f is fused vector (1 × 1186), p denotes features probability, and He represents entropy. This entropy-based feature selection method selects similar or highly related features from the fused vector. Unrelated features were removed, and appropriate features were considered for classification. In this proposed system, the appropriate feature vector is 1 × 1186. CNN classifier uses the selected features where the process of feature extraction and selection is shown in Figure 8. Furthermore, the feature vectors obtained by HOG and deep learning were fused to validate the proposed approach in this work.

The VGG19 architecture consisted of 16 layers of CNN, 3 connected layers with 1 final output layer for conducting SoftMax function. No changes in the number of connected layers and final layer are required to build the network architectures. In addition, 2 × 2-pixel windows with stride 2 were used for the max-pooling layer. The first two layers and third layer of the three fully connected layers provided 4096 features and 1000 channels, respectively. The final layer represents the output layer with two neurons (COVID-19 and normal).

### 3.7. Segmentation of the COVID-19-Affected Region

**Figure 9:**
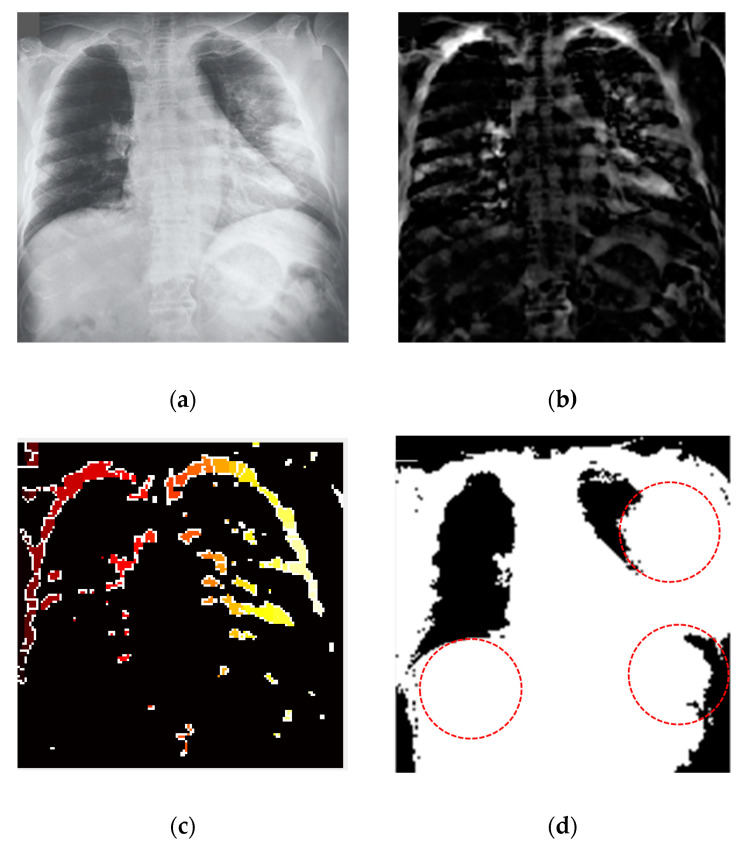
COVID-19 segmentation by using the watershed technique (**a**) applied anisotropic diffusion for filtering (**b**) adjusting the filtered image (**c**) watershed RBG image (**d**) fracture lung region caused by the coronavirus (COVID-19).

For biomedical image segmentation, the watershed technique [55] provides better results compared to the other techniques, such as Fuzzy-C means (FCM). The conventional FCM algorithm suffers from some weaknesses in terms of initializing clusters center or determining an optimal number of clusters and sensitiveness to noise [56]. FCM segmentation method cannot detect the fracture regions from the X-ray images affected by the COVID-19. However, watershed segmentation is a fast, simple and intuitive method, which provides closed contours, requires low computational time and produces a complete division of the image in separated regions. Segmentation was applied for the non-trivial task of separating the fracture lung regions from the X-ray images. A watershed segmentation technique was applied to segment the fracture regions of each image owing to its relatively less computational complexity and capability of providing high accuracy in segmentation. This method separated touching objects in an X-ray image and provided a complete division. Figure 9 presents different stages of image processing from filtering to segmentation of significant regions from the COVID-19-affected lung X-ray images.

## 4. Experimental Details and Results

### 4.1. Datasets and Overall Performance

To validate the framework developed for intelligent COVID-19 detection, this work used a total of 5090 chest X-ray images for training, testing and validation, as shown in Table 1 without data augmentation. In this study, the distribution of the data was laminated in order to mitigate the data disequilibrium issue. The validation images were taken from the training set, but the testing set was taken before training.

This research employed a total of 2489 normal and 1584 COVID-19-positive images for the training purpose. For the testing purpose, 622 normal images and 395 COVID-19-positive images were used. These testing images were not considered in the training dataset. This system also contained 70 validation images for both normal and COVID-19 classes. These validation images were taken from the training data set.

Three metrics, namely accuracy, specificity and sensitivity, were employed to measure the performance of the system developed for automatic COVID-19 detection from the chest X-ray images. Four different performance parameters, namely true-positive (TP), true-negative (TN), false-positive (FP) and false-negative (FN), were used to compute the metrics as defined by Equations (20)–(22).

(20) $$Accuracy\;\left(ACC\right)\;=\;\;\frac{TP+TN}{TP+TN+FP+FN}$$

(21) $$Specificity\;\left(SPEC\right)\;=\;\frac{TN}{TN+FP}$$

(22) $$Sensitivity\;\left(SEN\right)=\;\frac{TP}{TP+FN}$$

**Figure 10:**
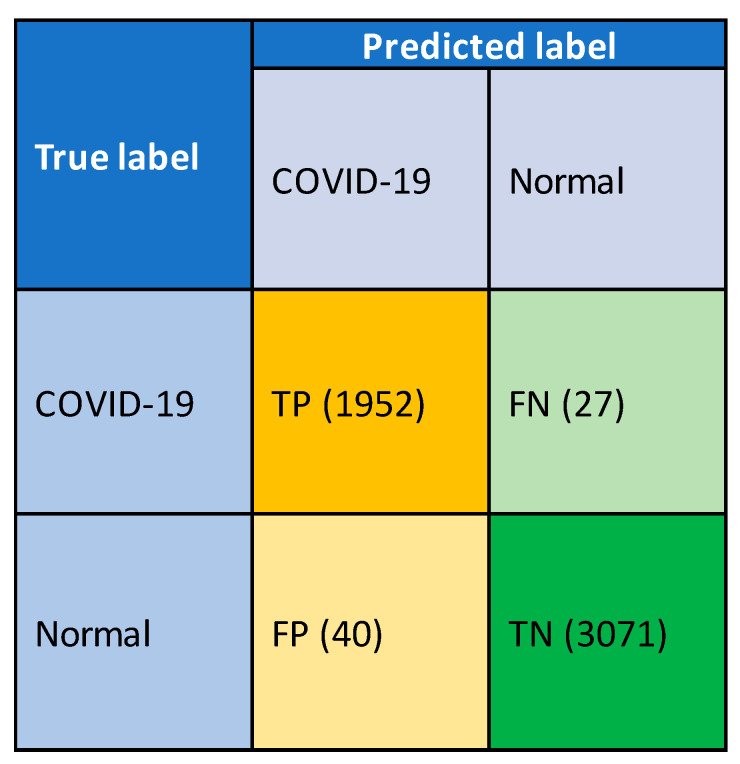
Confusion matrix with overall measured performance parameters during training.

Figure 10 presents the confusion matrix derived from the measured performance parameters for the overall system during the classification stage. During the course of the evaluation, the proposed method required labeled test data to validate its predicted output. The confusion matrix represents an overall system performance. The system cannot detect 27 COVID-19 positivity images and correctly detects 1952 COVID-19 positivity images out of 1979 images. The system cannot correctly detect 40 normal images and correctly detects 3071 normal images out of 3111 images.

### 4.2. Filtering Performance

**Figure 11:**
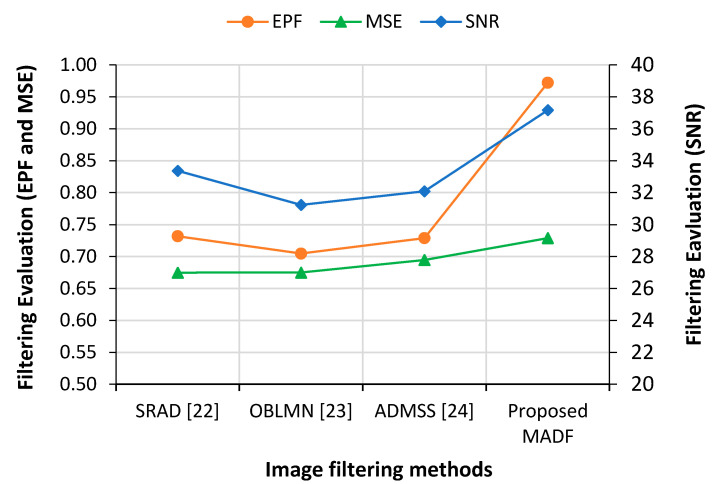
Performance comparison of modified anisotropic diffusion filtering (MADF) with other techniques in the literature.

The proposed method used COVID-19 X-ray images as test data with different speckles, noises and resolutions. To work with meaningful features, information preservation and noise reduction are the prerequisite conditions to fulfill. The current system used modified anisotropic diffusion filtering (MADF) at the image preprocessing stage. The performance measurement in MADF was assessed using three evaluation metrics, namely Signal-to-noise ratio (SNR), minimum square error (MSE) and edge preservation factors (EPF) [21]. Higher values of SNR and EPF represent more noise reduction and much edge details preservation, respectively. On the other hand, the minimum MSE value indicates less error between the input and filtered images. Classification models were run 10 times, and the highest values were reported for the performance metrics. Figure 11 presents the filtering performance using different evaluation metrics and comparison with other techniques available in the literature. It was clear that all the existing filtering techniques produced lower MSE values indicating that the proposed technique was only slightly worse than the existing techniques. On the other hand, the SNR and EPF values were comparatively much higher in the proposed filtering technique, demonstrating its superiority over the others.

### 4.3. Feature Extraction Performance

**Figure 12:**
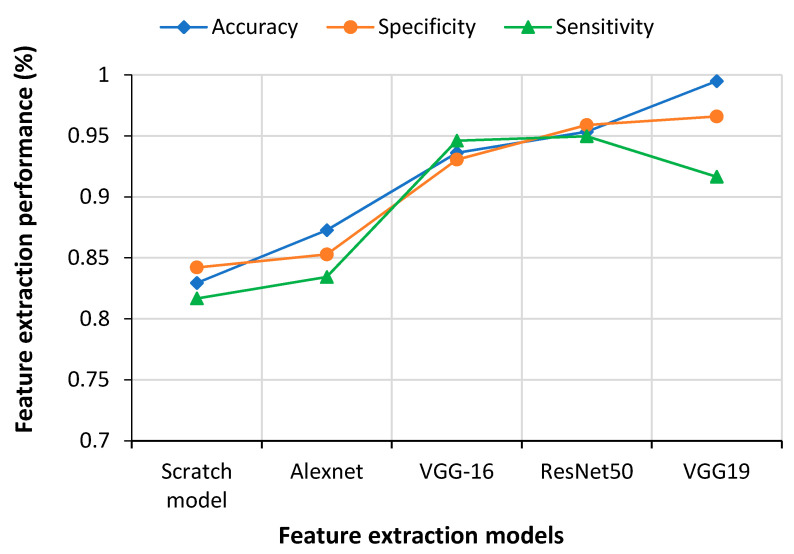
Performance measurement of different feature extraction models.

CNN used extracted features to train before classifying. Test features were also found from the test images using different pre-trained models to measure the performance of CNN models. Nowadays, CNN uses different pre-trained models like AlexNet, ResNet50, VGG-16, VGG-19 and ResNet50 to extract features from the training and test data sets. All these models produce similar results for standard training and testing data. Figure 12 presents a comparison of performance among different CNN models. It was apparent that VGG19, which was proposed in this work, achieved better accuracy and specificity than the other CNN models, although ResNet50 showed the best performance in terms of sensitivity.

However, the scratch model did not show satisfactory performance compared to the CNN models. Overall, ResNet50 and VGG19 models generated much better results than the VGG16 and AlexNet models.

### 4.4. Classification Performance

**Figure 13:**
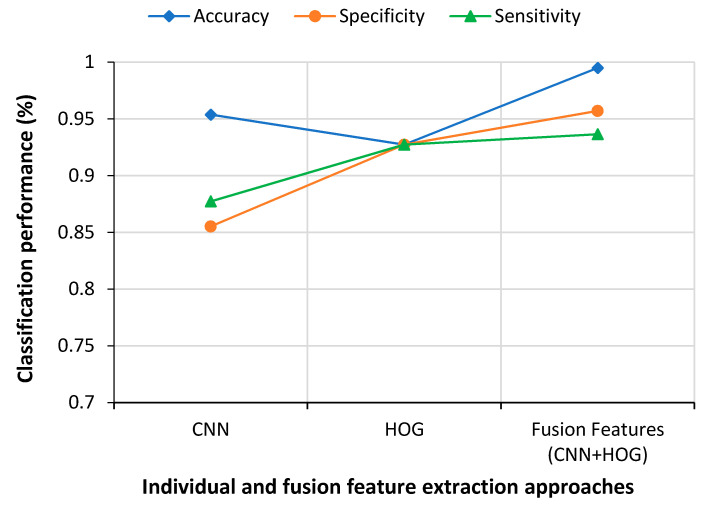
Comparative results of individual and fusion features.

This work proposed a fusion of feature vectors obtained by a combination of HOG and CNN techniques. This fusion vector was deliberated as the final input for the training and test datasets. Figure 13 presents a comparative study of different feature extraction approaches. The performances of different individual feature extraction techniques were less satisfactory than the fusion approach. This demonstrated that the proposed approach could classify COVID-19 cases more accurately than the single feature extraction approaches.

**Figure 14:**
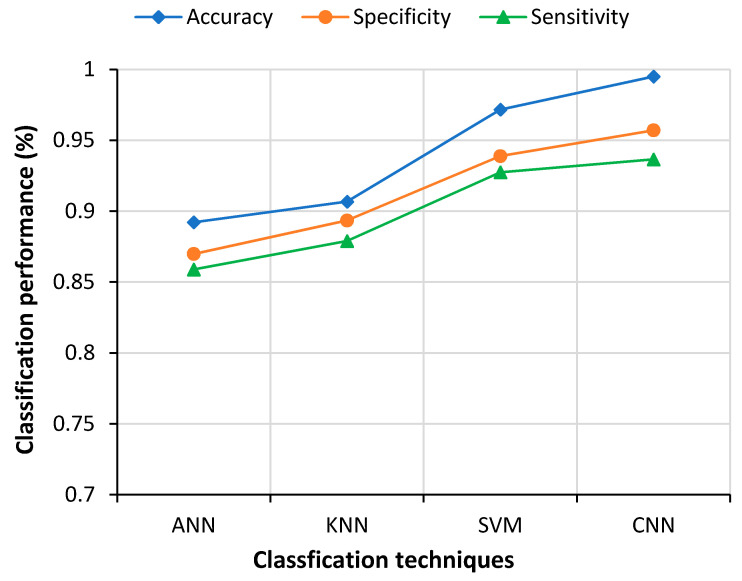
Comparative performance of different classifiers.

The final classification was also performed with other popular machine-learning methods, such as artificial neural network (ANN), support vector machine (SVM) and k-nearest neighbor (KNN), in addition to CNN. The fused feature vectors were fed to during the classification to find the better classifier. CNN clearly showed the best performance, as shown in Figure 14.

**Figure 15:**
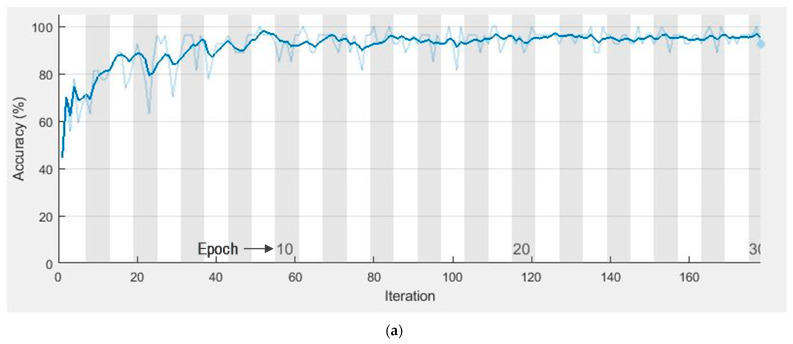

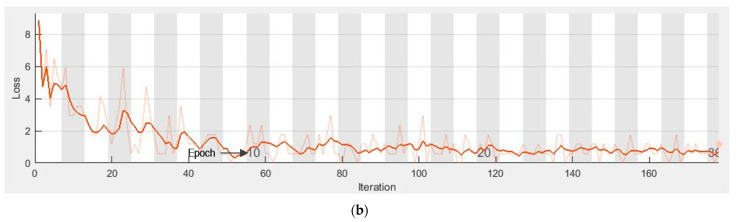
Learning curves (**a**) accuracy vs. number of epochs (**b**) loss vs. number of epochs.

Accuracy vs. epoch curve is plotted in Figure 15a. This showed clear evidence of no overfitting situation with very close training and accuracy curves. The learning rate starts from 0.001 with mini-batch sizes of 64 and 36 epochs. The loss curve depicted in Figure 15b indicated only a little amount of lost value.

## 5. Discussion

### 5.1. System Validation

To further validate the system’s performance, generalization and k-fold validation techniques were applied.

**Figure 16:**
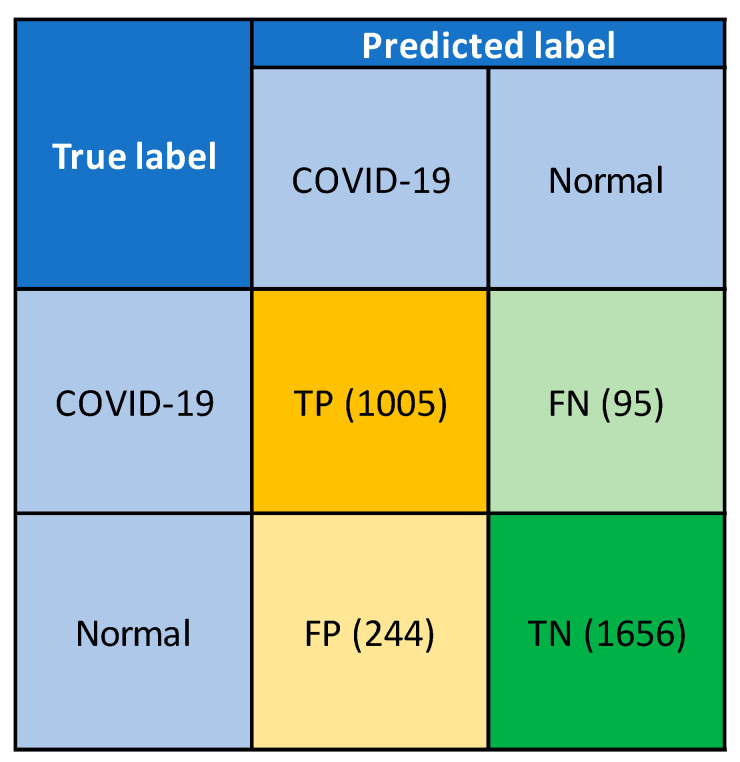
Confusion matrix from generalization.

Generalization is a term that measures a model’s ability to react to new data. After being trained, this approach can digest new data and make accurate predictions. The existing work faced difficulties in the X-ray image quality, which negatively influence the generalization performance. This research used the IEEE Data Port X-ray image dataset for generalization purposes, which was completely different from the training set [57]. This dataset was previously unsighted and unknown for this model. In generalization, 3000 images were used with two classes of the COVID-19 (1100 images) and normal (1900 images). The confusion metrics of the generalization results are presented in Figure 16. Among 1100 COVID-19 images, only 95 images are miss detected. The wrong detection rate was minimum for the COVID-19 chest X-ray images, which achieved a generalization accuracy of 93.75%. This indicated the image classification reliability of the system even with a completely new set of data.

K-fold cross-validation is primarily used in applied machine learning to estimate the performance of a model to make predictions on data not used during the training of the model [58]. A k-fold cross-validation technique was performed to gain further confidence in the experimental results. CNN classifier was trained by 5-fold cross-validation after feature extraction where feature vector was randomly distributed into 5 sub folds. Four sub folds were selected for the training dataset, and one single sub fold was selected for the testing dataset. Table 2 presents the different CNN classification results using individual and combined feature extraction methods. Single feature extraction methods provided less accuracy compared to the proposed fusion vector.

### 5.2. Comparative Analysis

Using the relevant datasets of chest X-ray images for the COVID-19 detection is a laborious task. The researchers used different preprocessing techniques, feature extraction techniques and classification methods [29,30,31,32,33,34,35,36,37,38,39]. Now, it is difficult to suggest a promising technique or combination of techniques that are more effective in diagnosing COVID-19 from the chest X-ray image. In most of the cases, an accuracy above 90% was reported, and from a statistical point of view, this a very high level of accuracy. However, the goal would be increasing the accuracy level as close to 100% as misdiagnosis, even in a small number of cases, is not quite acceptable.

The comparative analyses of related work are shown in Table 3. It is fairly clear that the proposed technique produced a better classification accuracy in detecting COVID-19 compared to the other techniques proposed in the literature. However, the study by Loye et al. [35] found a higher accuracy (100%) than this study. This could be due to a much lower number of images (69 COVID-19 and 79 normal images) used in their dataset for testing the system’s performance [59].

The proposed method employing feature fusion extracted by HOG and CNN (VGG19) showed better accuracy using CNN classifier. CNN provides a good result as a binary classifier for the chest X-ray dataset. However, when the classification was carried out with features extracted either by CNN or HOG individually, the accuracies were much lower compared to the reported values. HOG was the worst among the three, with an accuracy of 92.73%. Similarly, only SVM (97.16%) showed accuracy higher than 95% compared to the other two classification techniques, such as ANN (89.21%) and KNN (90.67%).

In this study, even though the image size was reduced by more than half of the original sizes, the system still demonstrated its robustness in correctly diagnosing COVID-19 cases. This could be attributed to the higher number of distinctive features obtained by the fusions of features extracted by HOG and CNN.

The proposed technique suggests a robust way in the feature extraction and noise removal phase. The proposed system used speckle-attacked and low-quality chest X-ray images in the testing phase. The MADF technique used in this work dispelled multiplicative speckle noise from the test images. It can effectively overcome the drawbacks of noisy images. Thus, the technique applied for preprocessing the input images facilitated more effective feature extraction and subsequently made a positive impact towards gaining a better classification performance. The results of the study demonstrated that the proposed feature fusion approach could diagnose COVID-19 very quickly and accurately and help the clinicians to take the necessary actions for saving the lives of COVID-19-affected people.

### 5.3. Limitations and Future Work

One of the limitations of this work was the imbalance of data in the datasets used for training and testing. In general, balanced data set with an equal number of normal and COVID-19 X-ray images makes the model building more comfortable, and the developed model can provide better prediction accuracy. Furthermore, the classification algorithm finds it easier to learn from a balanced dataset. Naturally, in any opensource database, the number of normal images would be higher than the COVID-19-positive images. As the images used in this study were taken from open-source databases, the imbalance in the training and testing data sets was obvious. However, the ratio between the number of normal and COVID-19 images was maintained at 1.57 in both the training and testing data sets in order to alleviate the data imbalance problem to some extent.

Although some of the studies in the literature used balanced data sets [60,64,65] for the COVID-19 detection using CNN on X-ray images, a large number of studies employed unbalanced data [30,31,32,35,36]. Although, Azemin et al. [32] and Minaee et al. [36] built CNN models with hugely imbalanced data with normal to the COVID-19 ratios of 37 and 28, respectively, the models still showed high-performance matrices. In comparison, the data sets in this study with a ratio of 1.57 make it only slightly imbalanced datasets. Therefore, it can be said that the imbalanced data are not the only factor that could affect the prediction accuracy; other factors, such as data set size, filtering technique, feature extraction technique, and the machine-learning algorithm used, should also be taken into consideration.

Another limitation of this study is the small number of X-ray images. New images are continuously being made available around the world. In order to further improve the robustness and accuracy of the model, a number of images will be added to the training data set. The trained model can be stored in the cloud. A mobile app can be developed to diagnose COVID-19 within seconds in order to reduce the workload of clinicians in the hospitals. The future work will focus on the fusion of HOG and CNN features for three-dimensional volume analysis. In addition, more efficient methods can be developed by exploring other fusion approaches to improve the result.

In order to respond to the global challenge of quickly identifying COVID-19, a large number of studies were carried out in 2020 by applying deep learning techniques on lung X-ray images. The studies varied significantly in terms of deep learning architecture, feature selection technique, number of images in the training and testing data sets, testing protocols, etc. However, the majority of the studies reported high classification performances in discriminating COVID-19 using different deep learning techniques. Concerns have been raised by Cohen et al. about the effectiveness in practical applications of the classification models with chest X-ray images [69]. Learning bias has been highlighted as one of the key challenges for the development of a robust and statistically stable deep learning model, which can confidently make the prediction for an unseen data set with similar values of the performance metrics. The bias could be due to a small number of the COVID-19 images with a publicly available large number of non-COVID-19 images [70]. Furthermore, the learning bias could be due to the source data set, meaning that the model could learn features that are related to the characteristics of the datasets (text label, scan setting, or similar age and sex) rather than the features specific to the disease impacting the overall validity of the model and its significance in clinical use [69,71]. Maguolo et al. demonstrated that, interestingly, even without containing a major part of the lungs in the X-ray images within a dataset could produce similar classification results compared to the dataset of X-ray images, including lungs [71]. Designing a fair testing protocol could be highly challenging when different datasets were merged with large differences among them. This could generate misleading results. Therefore, assessing the validity of the available testing protocol must be carefully dealt with.

In this study, despite taking some preventative measures to avoid bias, such as using relatively large data set (3111 non-COVID and 1979 COVID-19 images) without huge data imbalanced, preprocessing of images (e.g., removing text labels) to remove dataset-dependent features, extracting features by two techniques and feature fusion and testing with a set of data different from the training set, still, the trained model would not be completely free from the learning bias. Yasar et al. compiled results for the COVID-19 detection by recent studies using the X-ray images and found that seven out of sixteen (44%) studies used k-fold validation [59]. This represented that k-fold was a popular validation technique. In this study, during k-fold validation, as the images were taken from the same training and testing datasets, unlike the generalization technique where new data set were used for the validation, it provided accuracy close to the testing accuracy. This supported the argument that k-fold validation could be an unfair testing protocol. Although k-fold cross validation balanced out the predicted features’ classes with the unbalanced dataset used in this study to a certain extent, still innovative and effective testing protocols must be sought to make sure the developed model would be free of any learning bias for an accurate prediction of the outcome. This could be possible in the future when the continuous effort of collecting X-ray images will create a large balanced dataset to determine if deep learning can provide a solution in the fight against the COVID-19 pandemic.

## 6. Conclusions

The coronavirus pandemic has stretched the healthcare systems in every country in the world to its limit as they had to deal with a large number of deaths. Early detection of the COVID-19 in a faster, easier, and cheaper way can help in saving lives and reduce the burden on healthcare professionals. Artificial intelligence can play a big role in identifying COVID-19 by applying image processing techniques to X-ray images. This work designed and developed an intelligent system for the COVID-19 identification with high accuracy and minimum complexity by combining the features extracted by histogram-oriented gradient (HOG) features and convolutional neural network (CNN). Suitable feature selection and classification are absolutely vital in the COVID-19 detection using chest X-ray images. Chest X-ray images were entered into the system in order to produce the output of the marked lung significant region, which was used to identify COVID-19. The proposed feature fusion system showed a higher classification accuracy (99.49%) than the accuracies obtained by using features obtained by individual feature extraction techniques, such as HOG and CNN. CNN produced the best classification accuracy compared to the other classification techniques, such as ANN, KNN and SVM. Furthermore, the proposed fusion technique was validated with higher accuracies using generalization and k-fold validation techniques.

## Figures and Tables

**Table 1:** Number of images in normal and COVID 19 categories used in training, validation, and testing phases without data augmentation.

Data Sets	Number of Images		Ratio of Normal to the COVID-19 Images
	Normal	COVID-19	
Training	2489	1584	1.57
Validation	70	70	1.0
Testing	622	395	1.57

**Table 2:** Overall classification accuracy measured using 5-fold cross-validation.

Feature Extraction Methods	Fold 1	Fold 2	Fold 3	Fold 4	Fold 5	Mean Accuracy
HOG	0.8732	0.8789	0.8741	0.8675	0.8730	0.8734
CNN	0.9378	0.9367	0.9387	0.9367	0.9321	0.9364
Proposed fusion (HOG+CNN)	0.9856	0.9847	0.9813	0.9827	0.9833	0.9836

**Table 3:** Comparison among existing methods in the COVID-19 detection.

References	Dataset	Methods	Accuracy
Ahammed et al. [29]	2971 chest X-ray images (COVID-19 = 285, normal = 1341, pneumonia = 1345)	CNN	94.03%
Chowdhury et al. [30]	2905 chest X-ray images (COVID-19 = 219, normal = 1341 and pneumonia = 1345)	Parallel-dilated CNN	96.58%
Abbas et al. [31]	196 CXR images (COVID-19 = 105, normal = 80, and SARScases = 11)	Deep CNN (DeTraC)	93.1%
Azemin et al. [32]	5982 (COVID-19 = 154 and normal = 5828)	ResNet-101 CNN	71.9%
El-Rashidy et al. [33]	750 chest X-ray images(COVID-19 = 250 and normal = 500)	CNN/ConvNet	97.95%
Khan et al. [34]	1057 X-ray images (COVID-19 = 195 and normal = 862)	VGG16+VGG19	99.3%
Loey et al. [35]	307 X-ray images (COVID-19 = 69, normal = 79, Pneumonia_bac = 79 and Pneumonia_vir = 79)	AlexNet+ Googlenet+Restnet18	100%
Minaee et al. [36]	50,184 chest X-ray images (COVID-19 = 184 and normal = 5000)	ResNet18 + ResNet50 + SqueezeNet + DenseNet-121	98%
Sekeroglu et al. [37]	6100 X-ray images (COVID-19 = 225, normal = 1583 and pneumonia = 4292)	CNN	98.50%
Wang et al. [38]	18,567 X-ray images (COVID-19 = 140, normal = 8851 and Pneumonia = 9576)	ResNet-101 + ResNet-152	96.1%
Panwar et al. [60]	284 images (COVID-19 = 142 and normal = 142)	Convolutional neural network (nCOVnet)	88.1%
Ozturk et al. [61]	625 images (COVID-19 = 125 and normal = 500)	Convolutional neural network (DarkNet)	98.08%
Khan et al. [62]	594 images (COVID-19 = 284 and normal = 310)	Convolutional neural network (CoroNet (Xception))	99%
Apostolopoulos and Mpesiana [63]	728 images (COVID-19 = 224 and normal = 504)	Transfer learning with convolutional neural networks(VGG19, MobileNet v2, Inception, Xception, InceptionResNet v2)	96.78%
Mahmud et al. [64]	610 images (COVID-19 = 305 and normal = 305)	Transfer learning with convolutional neural networks(stacked MultiResolution CovXNet)	97.4%
Benbrahim et al. [65]	320 images (COVID-19 = 160 and normal = 160)	Transfer learning with convolutional neural networks(Inceptionv3 and ResNet50)	99.01%
Martínez et al. [66]	240 images (COVID-19 = 120 and normal = 120)	Convolutional neural network (Neural Architecture Searchnetwork (NASNet))	97%
Toraman et al. [67]	1281 images (COVID-19 = 231 and normal = 1050)	Convolutional neural network (CapsNet)	97.24%
Duran-Lopezet al. [68]	6926 images (COVID-19 = 2589 and normal = 4337)	Convolutional neural network	94.43%
Proposed Method	5090 chest X-ray images (COVID-19 = 1979 and normal = 3111)	Fusion features (CNN+HOG) + VGG19 pre-train model	99.49%
